# No Easy Answers: Avoiding Potential Pitfalls of De‐implementation

**DOI:** 10.1002/ajcp.12298

**Published:** 2018-12-14

**Authors:** Rogério M. Pinto, Susan S. Witte

**Affiliations:** ^1^ School of Social Work University of Michigan‐Ann Arbor Ann Arbor MI USA; ^2^ School of Social Work Columbia University New York NY USA

**Keywords:** De‐implementation pitfalls, Politics, HIV‐prevention, Pre‐exposure prophylaxis

## Abstract

In 2012, the Centers for Disease Control and Prevention (CDC) began to de‐emphasize and de‐implement multiple evidence‐based HIV prevention practices that had been around for 20 years, thus changing the scope of implementation across the globe. The authors provide evidence how existing interventions (e.g., CDC HIV interventions) may influence implementation of interventions that came after the program was discontinued. De‐implementation is an ecological event that influences, and is influenced by, many parts of a system, for instance, implementation of one type of intervention may influence the implementation of other interventions (biomedical and/or behavioral) after a long‐running program is discontinued. Researchers and policy makers ought to consider how de‐implementation of behavioral interventions is influenced by biomedical interventions mass‐produced by companies with lobbying power. The scientific study of de‐implementation will be inadequate without consideration of the political climate that surrounds de‐implementation of certain types of interventions and the promotion of more‐profitable ones.

## Highlights

- Implementation of one type of intervention (behavioral) influences implementation of other types (biomedical) interventions.
- The surge of biomedical interventions influenced de‐implementation of behavioral interventions.
- Political climate and community exclusion from implementation decision‐making may lead to early de‐implementation.

In “Letting Go: Conceptualizing Intervention De‐implementation in Public Health and Social Service Settings,” the authors offer three criteria for identifying interventions appropriate for de‐implementation: they must be (a) harmful or not effective, (b) not the most effective or efficient to provide, or (c) no longer necessary (McKay, Morshed, Brownson, Proctor, & Prusaczyk, 2018). The authors also describe scenarios in which the de‐implementation process may take place, examining frameworks that may help us conceptualize that process.

We recognize the importance of works articulating criteria under which de‐implementation decisions are made. De‐implementation is an implicit part of the implementation process and requires the increased attention McKay et al. (2018) provide. However, we are concerned that the authors highlight examples of de‐implementation without delving more deeply into the more‐complex issues inherent in the process, including the importance of community input and the recognition that it is a dynamic, ecological process (Kelly, 2006; Trickett, 2009). We examine these issues as they pertain to the Diffusion of [HIV‐Prevention] Effective Behavioral Interventions (DEBI) program of the Centers for Disease Control and Prevention (CDC).

## Implementation of One Type of Intervention Influences Implementation of Other Types of Interventions and Services after a Program Is Discontinued

In McKay et al. (2018), DEBI interventions are discussed as an example of the de‐implementation criterion for interventions that are not the most effective or efficient to provide or are no longer necessary (“low‐value” interventions). The DEBI program was built over the course of more than 20 years, during which the CDC funded the development, dissemination, and implementation (Dworkin, Pinto, Hunter, Rapkin, & Remien, 2008) of nearly 30 evidence‐based, behavioral HIV‐prevention interventions. In 2008, more than a decade into the DEBI program, the *American Journal of Community Psychology* published a paper noting that “this is a particularly timely juncture in which to critically reflect on the extent to which known principles of community collaboration have guided key processes associated with the DEBI rollout” (Dworkin et al., 2008, p. 3). In that paper, the authors reviewed the evidence and posited that important considerations had been overlooked in the rollout of the DEBI program, including community perceptions of a top‐down mode of dissemination and the extent to which local innovations were being embraced, bolstered, or eliminated. Since 2012, with the advent of biomedical interventions, considered to be more cost‐effective, shifts in priorities took place in order to refocus attention on “high‐impact prevention” (HIP), targeting individuals living with HIV infection and their partners. There has been an enormous effort by the CDC to deemphasize and de‐implement most of its behavioral evidence–based HIV‐prevention interventions. This de‐implementation by the CDC of more than two‐thirds of its DEBIs has completely changed the scope of implementation in HIV prevention in the United States and across the globe, and thus needs further exploration. This de‐implementation was also largely uninformed by the communities of HIV services administrators, providers and clients, all who were most directly affected by the process.

McKay and colleagues (2018) acknowledge that “it is important to understand the outcomes of de‐implementation…. It will also be valuable to understand some of the ancillary benefits that may have occurred because of implementing interventions” (p. 10).

In our published study (Pinto, Witte, Filippone, Choi, & Wall, 2018), we contributed to a better understanding of the outcomes of de‐implementation. Using data from 379 HIV service providers, working at 36 agencies in New York City, we found significant associations between exposure to HIV‐prevention interventions (defined as *providers of social and public health services in agencies providing EBIs*) and the frequency with which these providers made links to high‐impact services. We found that providers who are exposed to *any* HIV‐prevention interventions more frequently link clients to HIV, HEP‐C, and STI testing, to primary care, and to drug‐treatment and mental health services, all of which are evidence‐based practices shown to help decrease the spread of HIV. In other words, exposure to many of the interventions, now de‐implemented, may have contributed to the relative success of newly implemented high‐impact interventions. Our findings also suggest a dose effect, with exposure to more EBIs resulting in more links to high‐impact interventions. If exposure to EBIs increased the likelihood of links being made to HIV primary care and other “higher‐value” programs, there exists the potential for de‐implementation to have a long‐term inadvertent effect, potentially negative, on the implementation of future innovations.

These findings lend support to the innovative concept of “exposure” to evidence‐based practices, and thus shed light on how a system‐level program such as DEBI can influence adoption of evidence‐based services (e.g., HIV testing) under a subsequent program (e.g., HIP). “Exposure” suggests the potential for a staged approach to implementation of HIV‐prevention innovations, and perhaps to innovations in other areas of social and public health. Providers were initially exposed to DEBI and were required to offer multiple EBIs; then, after the emergence of HIP, they were required to offer high‐impact innovations. But this historical development was not without unwanted consequences. For instance, de‐implementation/de‐adoption of RESPECT, an individual‐level EBI for reducing HIV risks, has weakened collaborative interagency relationships and caused frustration among staff members (McKay, Dolcini, & Hoffer, 2017). There may be many more examples that have not been studied; further research is needed to examine the individual effects of de‐implementation of long‐running practices.

## De‐implementation of Behavioral Interventions and the Surge of Biomedical Ones

In 2012, antiretroviral therapy surfaced as the leading approach for HIV treatment and prevention. At that time, pre‐exposure prophylaxis (PrEP), a daily oral dose of antiretroviral therapy prescribed to individuals at risk for HIV infection, emerged as a major HIV‐prevention tool. PrEP was shown to reduce the risk of HIV acquisition by 73% among adult men who have sex with men (MSM) and transgender women, with greater efficacy (up to 99%) for individuals with higher rates of adherence (Anderson et al., 2012; Donnell et al., 2014; Grant et al., 2010). Here, we see the appearance of a pharmaceutical intervention driving a policy of de‐implementation when none of the three categories developed by McKay et al. (2018) would apply. In practice, behavioral interventions were largely discontinued while PrEP became a leading form of HIV prevention, but not because HIV behavioral interventions were (a) harmful or no longer effective, (b) the least effective or efficient to provide, or (c) no longer necessary. Indeed, for at‐risk individuals to adhere consistently to PrEP, they need to have the same cognitive skills—knowledge, attitudes, self‐efficacy—that result from the HIV‐prevention behavioral interventions that are, in many cases, no longer available.

The fast shift from behavioral to medical interventions has inherent barriers to implementation (Pinto, Berringer, Melendez, & Mmeje, 2018), and the surge of HIV‐prevention biomedical interventions has exacerbated already existing racial and gender disparities in HIV prevention and treatment—for instance, disproportionately low PrEP uptake among black MSM (Eaton, Driffin, Bauermeister, Smith, & Conway‐Washington, 2015), who represent a historically underserved population. In addition, research regarding low access, uptake, and adherence to PrEP has shown breakdowns in healthcare systems implementing PrEP, a lack of medical providers' knowledge or willingness to prescribe PrEP (Krakower, Ware, Mitty, Maloney, & Mayer, 2014; Petroll et al., 2017), and unfavorable community attitudes about PrEP (Krakower et al., 2012; Liu et al., 2008). These barriers reflect those we have witnessed for the past 20 years in relation to CDC DEBIs, which have been discussed at length (Dworkin et al., 2008; Pinto, Berringer, et al., 2018; Pinto, Witte, et al., 2018). These barriers to intervention implementation also reflect both the political climate and community exclusion from implementation decision‐making.

## Political Climate and Community Exclusion from Implementation Decision‐Making

Despite the call to arms by Dworkin et al. (2008) noted earlier regarding DEBI implementation – the lack of attention to community perceptions in the implementation process – we see that these same considerations were overlooked in the process of de‐implementation.

In response to the research described above and the need for more cost‐effectiveness, in 2015 the CDC awarded $216 million to 90 community‐based agencies to deliver HIV‐prevention programs to populations most vulnerable to HIV infection and transmission—certain racial and ethnic groups, MSM, transgender individuals, and individuals who used injectable drugs (Centers for Disease Control and Prevention, 2015). That same year, President Obama signed Executive Order 13707, *Using Behavioral Science Insights to Better Serve the American People*, encouraging policy makers to “identify policies, programs, and operations where applying behavioral science insights may yield substantial improvements in public welfare, program outcomes, and program cost effectiveness” (White House, 2015).

This suggests that DEBI de‐implementation has followed political reality; the question is in whose interest (or at whose expense) such cost‐effectiveness may be gained. While the CDC de‐implementation may have taken place for cost‐efficiency or program‐value reasons, we may have lost the relative benefits of continued implementation of behavioral interventions to reach marginalized groups who are at risk for new HIV infections and who may not be accessing important HIV‐prevention tools such as PrEP. We do not yet know the long‐term effects, and agree that de‐implementation must be monitored for potential harmful effects.

Community exclusion from decision‐making processes related to scientific research and implementation of its products has been helpful in explaining why community members (who can most benefit from research innovations) and the practitioners who deliver those innovations are wary of scientific knowledge and thus may reject its products (e.g., EBIs). Community exclusion also helps explain why communities, including consumers, providers, and agency administrators, are often suspicious of scientific knowledge and find its products inadequate and sometimes ineffective in the effort to address specific complexities of local populations (Dworkin et al., 2008; Pinto, 2012). As researchers, we are also aware of the severe limitations of EBIs when considering program fit. Most studies establishing efficacy of EBIs do not adequately control for nonspecific factors such as attention and treatment intensity, and have thus failed to assess specific mediators of change (see e.g., Jensen, Weersing, Hoagwood, & Goldman, 2005). Community skepticism is a fair and authentic response. Consideration of these additional factors is necessary if we are to effectively document, manage, and advance the science of dissemination and technology transfer in centralized prevention efforts within and outside HIV/AIDS.

## Conclusion

De‐implementation of evidence‐based practices is an ecological event that may influence, and be influenced by, many parts of a system (Trickett, 2009). As such, it is much more complex than the three criteria described in McKay et al. (2018). Perhaps, in addition to criteria focused on the interventions themselves, an additional set of criteria is needed to account for the processes inherent in de‐implementation. The work of Wang, Maciejewski, Helfrich, and Weiner (2018), in which de‐implementation is represented using four types of change, may be a critical addition to complement and strengthen the work of McKay et al. (2018). That approach recognizes the different challenges and dynamics that can occur in de‐implementation. Wang and colleagues offer a typology of de‐implementation that describes four types of change: partial reduction, complete reversal, substitution with related replacement, and substitution with unrelated replacement of existing practice. This approach is more consistent with organization‐based practices that are not necessarily discrete programs or packages, but rather practices that may be changed to facilitate closer collaboration with staff and community members.

Future criteria and/or typologies may benefit from, and thus consider, the available evidence that implementation of one type of intervention may influence the implementation of other interventions (biomedical and/or behavioral) and services after a long‐running program is discontinued. Researchers and policy makers have much to gain by considering how de‐implementation of behavioral interventions is influenced by the surge of biomedical interventions that are developed and mass‐produced by companies with lobbying power that greatly surpasses that of behavioral researchers. Finally, the scientific study of de‐implementation will be inadequate without full consideration of the political climate that surrounds the de‐implementation of certain types of interventions and the promotion of more‐profitable ones. Here, genuine inclusion of community in decision‐making about implementation may imbue de‐implementation criteria with the opinions and lived experiences of those who may benefit the most from more‐effective interventions.
